# Measuring administrative integration of disease control programmes within health systems: a case study of HIV monitoring and evaluation in South Africa

**DOI:** 10.1186/1472-6963-14-S2-P62

**Published:** 2014-07-07

**Authors:** Mary Kawonga, Sharon Fonn, Duane Blaauw

**Affiliations:** 1 Department of Community Health, Wits School of Public Health, Faculty of Health Sciences, University of the Witwatersrand, Johannesburg, South Africa; 2 Wits School of Public Health, Faculty of Health Sciences, University of the Witwatersrand, Johannesburg, South Africa; 3 Centre for Health Policy, Wits School of Public Health, Faculty of Health Sciences, University of the Witwatersrand, Johannesburg, Johannesburg, South Africa

## Background

In South Africa, integrating the management of health services at a decentralized district level within the authority of general health service (GHS) managers is a reform priority. The reforms promote administrative integration, i.e. transfer of administrative authority over disease control programmes (DCPs) from DCP managers to GHS managers and re-defining DCP manager roles from implementation to specialist support. Historically DCP activities at district level fall under the control of DCP managers and dedicated programme monitoring and evaluation (M&E) systems limit GHS managers’ access to data that they need for integrated management. Given the reform focus on administrative integration, research is needed to measure the extent to which GHS managers exercise administrative authority over DCP functions. This study addresses this gap, using the HIV programme M&E function as an exemplar.

## Materials and methods

This study was conducted in two of South Africa’s nine provinces, involving interviews with 51 GHS and DCP managers. We adapted and applied the concept of ‘decision-space’ (traditionally used to measure transfer of administrative authority from higher to lower level managers). We defined ‘exercised authority’ over the HIV M&E function as performance of tasks to: a) oversee the production of HIV information (HIV data collection, collation and analysis), and b) use HIV information for monitoring services. Participants reported whether they performed specific tasks within each M&E domain (data collection, collation, analysis and use). Responses were scored within each domain and summed scores categorised as ‘low’, ‘medium’, or ‘high’ degree of exercised authority. We applied ordinal logistic regression to evaluate associations between various variables (actor type [DCP or GHS], capacity [training and experience] and HIV M&E knowledge) and the degree of exercised authority. We also assessed whether DCP actors play expected specialist support roles.

## Results

Relative to DCP managers, GHS managers had lower M&E knowledge and exercised greater authority over the production of HIV information but less over using HIV information. Higher HIV M&E knowledge was associated with higher degrees of HIV information use. DCP and GHS manager roles overlap; few DCP managers played their expected specialist support role.

## Conclusions

There has been transfer of authority for overseeing the production of HIV information from DCP to GHS managers, but DCP managers still control the use of HIV information and rarely play expected specialist support roles. Actions are needed to integrate the use of DCP information within GHS managers’ roles.

